# Experiences of stigma in healthcare settings among adults living with HIV in the Islamic Republic of Iran

**DOI:** 10.1186/1758-2652-13-27

**Published:** 2010-07-22

**Authors:** Fatemeh Rahmati-Najarkolaei, Shamsaddin Niknami, Farkhondeh Aminshokravi, Mohsen Bazargan, Fazlollah Ahmadi, Ebrahim Hadjizadeh, Sedigheh S Tavafian

**Affiliations:** 1 Health Education Department, Tarbiat Modares University, Tehran, Iran; 2 Department of Family Medicine, Charles Drew University of Medicine and Science, Los Angeles, California, USA; 3 Department of Nursing, Tarbiat Modares University, Tehran, Iran; 4 Department of Biostatistics, Tarbiat Modares University, Tehran, Iran

## Abstract

**Background:**

People living with HIV (PLHIV) sometimes experience discrimination. There is little understanding of the causes, forms and consequences of this stigma in Islamic countries. This qualitative study explored perceptions and experiences of PLHIV regarding both the quality of healthcare and the attitudes and behaviours of their healthcare providers in the Islamic Republic of Iran.

**Methods:**

In-depth, semi-structured interviews were held with a purposively selected group of 69 PLHIV recruited from two HIV care clinics in Tehran. Data were analyzed using the content analysis approach.

**Results and discussion:**

Nearly all participants reported experiencing stigma and discrimination by their healthcare providers in a variety of contexts. Participants perceived that their healthcare providers' fear of being infected with HIV, coupled with religious and negative value-based assumptions about PLHIV, led to high levels of stigma. Participants mentioned at least four major forms of stigma: (1) refusal of care; (2) sub-optimal care; (3) excessive precautions and physical distancing; and (4) humiliation and blaming. The participants' healthcare-seeking behavioural reactions to perceived stigma and discrimination included avoiding or delaying seeking care, not disclosing HIV status when seeking healthcare, and using spiritual healing. In addition, emotional responses to perceived acts of stigma included feeling undeserving of care, diminished motivation to stay healthy, feeling angry and vengeful, and experiencing emotional stress.

**Conclusions:**

While previous studies demonstrate that most Iranian healthcare providers report fairly positive attitudes towards PLHIV, our participants' experiences tell a different story. Therefore, it is imperative to engage both healthcare providers and PLHIV in designing interventions targeting stigma in healthcare settings. Additionally, specialized training programmes in universal precautions for health providers will lead to stigma reduction. National policies to strengthen medical training and to provide funding for stigma-reduction programming are strongly recommended. Investigating Islamic literature and instruction, as well as requesting official public statements from religious leaders regarding stigma and discrimination in healthcare settings, should be used in educational intervention programmes targeting healthcare providers. Finally, further studies are needed to investigate the role of the physician and religion in the local context.

## Background

Stigma affects the lives of individuals infected with HIV [1]. Particularly, disadvantaged people living with HIV (PLHIV) experience discrimination in their interactions with the healthcare system [2]. Stigma is considered to be a major barrier to effective responses to the HIV epidemic [3].

More than four decades ago, Goffman (1963) defined stigma as "an attribute that is deeply discrediting", and proposed that the stigmatized person is reduced "from a whole and usual person to a tainted, discounted one" [4] (p. 3). Even though this construct has generated extensive theoretical and empirical research [5], there is, as yet, no solid, common theoretical perspective on stigma [6].

Deacon (2006) constructed a sustainable theory of health-related stigma that brought together both the individual and social dimensions of this complex phenomenon that may facilitate interventions against health-related stigma. Deacon argues that stigma comes about in a social process during which the following occurs: illness is perceived as controllable or preventable, and caused by identifiable "immoral" behaviours. These behaviours are associated with certain groups that "carry" the illness, which draws on existing social constructs of the "other", who are consequently blamed for becoming infected.

These "others" experience status loss from the projection of blame, and may become disadvantaged as a result [6]. The internalization of this "blame" and the perceived "status loss" by stigmatized (HIV-infected) persons, combined with objective assessments of the infected person's day-to-day experiences with structural discrimination (institutional practices that disadvantage stigmatized persons) [7,8], together may create, in the stigmatized person's viewpoint, a perception of the healthcare system as intolerant and inaccessible [9].

Numerous studies have documented the attitudes of healthcare providers toward PLHIV [10-15]. Although the literature characterizes the attitudes and behaviours of healthcare providers as positive and respectful, many studies also report poor communication between patients and healthcare providers [16], which functions as a major barrier in providing proper care for these patients [17]. Few studies in the international body of literature explore the experiences of PLHIV in the context of the healthcare system. One recent cross-sectional study, conducted among a sample of 202 PLHIV in Los Angeles County, US, demonstrated that in a diverse and under-served sample of PLHIV, poor access (self-reported) to medical care is strongly associated with experiencing HIV stigma.

The effect of perceived discrimination or internalized HIV stigma on access to care, regular HIV care, and adherence to treatment need further attention [9]. Research suggests that exploring the experiences of PLHIV with the healthcare system may not only enhance the quality of care that patients receive, but could also improve quality of life for PLHIV [18]. In addition, the inter-relationship between stigma and other factors known to be associated with adherence to medical treatment among PLHIV needs to be further demarcated in future studies in order to identify targets for successful intervention programmes [9].

An increasing number of countries in the Middle East, North Africa and Asia, including those with Muslim majorities, have experienced or are at risk for HIV epidemics [19]. HIV transmission and occurrence of AIDS in the Middle East is increasing, while access to HIV care and antiretroviral therapy in the region lags behind access in most low- to middle-income countries [20].

Religious constraints on sexuality may have consequences for the transmission of sexually transmitted infections [21]. It has also been reported that Muslim countries have a lower prevalence of HIV than non-Muslims countries. Gray and colleagues (2004) have documented that in 38 sub-Saharan African countries, the percentage of a Muslim population negatively predicted HIV prevalence, indicating a lower HIV prevalence in regions with higher Muslim populations [21]. It has been suggested that even though HIV has traditionally been the subject of stigma worldwide, this stigma is more intense in Muslim countries [22].

In a recent comprehensive examination of literature, Nyblade and her colleagues (2009) provide a review of the various forms and causes of stigma documented in healthcare setting across regions [23]. Studies in Ethiopia, India, Senegal, Indonesia, Botswana, Jamaica, South Africa, Zambia, Viet Nam, Nigeria, Kenya, Thailand, Bali, and Tanzania reveal that "there are three main immediately actionable causes of HIV-related stigma in health facilities: lack of awareness among health workers of what stigma looks like and why it is damaging; fear of casual contact stemming from incomplete knowledge about HIV transmission; and the association of HIV with improper or immoral behaviour" [23].

In Iran, the first case of HIV was reported in 1987, and was followed by a rapid increase in the number of cases. Currently, the HIV prevalence rate in Iran is estimated at 0.2%. In 2007, there were officially 15,587 Iranians living with HIV, 14,702 (94.3%) of whom were male and 885 (5.7%) of whom were female. Over the 20-year surveillance period, the rate of HIV infections diagnosed annually among Iranian citizens gradually increased, and over the period 1997 to 2004, went from 1.38 to 4.6 cases per 100,000 people per year [24].

While only 10% of PLHIV in Iran have been infected as a result of sexual contact [25,26], sexually transmitted HIV infection is rapidly increasing, which represents a shift in the mode of transmission from drug use to sexual behaviours [27-29]. As a country that has established a national HIV treatment system, including 150 testing sites and a free needle-exchange programme, Iran is perceived by many as a leader in HIV prevention in the Middle East [30]. In 2005 the Iranian postal service unveiled a stamp emblazoned with a red ribbon for AIDS awareness. In 2006 there was an AIDS awareness concert and in 2007 school children created a 150 foot long painting to promote AIDS prevention and awareness.

In Iran, a limited number of studies have been conducted to assess the knowledge and perspectives of healthcare providers towards HIV infection [31-35]. These studies have reported that most healthcare providers hold positive attitudes towards patients with HIV; however, in practice they prefer not to care for these patients for fear of becoming infected with HIV [35]. Studies that explore how PLHIV perceive discrimination may improve our understanding of the nature, extent and consequences of discrimination in patient-provider interactions [36].

Assessing how PLHIV perceive and experience the behaviours and attitudes of providers is important; the subjective beliefs that PLHIV hold about the situations in which they find themselves may or may not correspond to objective reality, but are nonetheless powerful forces with real consequences for their health-seeking behaviours. To address these questions and the gap in HIV and AIDS research in Iran, this qualitative study explored the perceptions and experiences of PLHIV regarding the attitudes and behaviours of healthcare providers and quality of healthcare.

## Methods

### Sample

In this qualitative study, participants were recruited from two HIV care clinics affiliated with the Medical School of Tehran University and University of Medical Sciences, as well as the Department of HIV Infection of Imam Khomeini Hospital. Currently, there are three large HIV care clinics in the greater metropolitan area of Tehran that provide comprehensive care, including medical and psychological care. PLHIV receive referrals for drug rehabilitation, ophthalmological, gynaecological, gastroenterological, dental and other specialty care. However, the specialty care facilities are not specific to HIV patients. The study sites record serving approximately 4500 PLHIV annually.

The sampling method involved the first author of this study approaching potential participants waiting in line to receive their clinical care. Seventy-six potential participants were invited to participate in the study. Of these, seven were not enrolled into the study due to lack of interest and other clinic-related reasons. A total of 69 participants then consented and were enrolled into the study.

A convenience sampling method was used to choose potential eligible participants, and then a purposeful sampling method was used to achieve data saturation. Purposive samples are the most commonly used form of non-probabilistic sampling for conducting qualitative research. In order for analytic generalizations to be richer, qualitative researchers typically rely on the concept of "saturation", or the point at which no new information or themes are observed in the data [37,38].

The first author of this study systematically reviewed the degree of data saturation over the course of thematic analysis. We found that saturation occurred within the first 60 interviews; however, to be on the safe side, we collected data from an additional 10 participants. This study reports data from 69 semi-structured interviews with PLHIV who provided informed consent. The interviews were conducted between March 2008 and January 2009.

### Data collection

The main author conducted all the one-on-one interviews. Semi-structured and in-depth interviews were conducted in private rooms, recorded on audiotapes, and transcribed verbatim. Prior to conducting the interviews, three qualitative research experts and five non-participants reviewed the interview items to assess the survey instrument. The interview consisted of open-ended questions, including the following: How do you feel about your healthcare providers? Do you have any problems with them? How did your disease influence the care you received from healthcare providers? What do you think about your interaction with them?

At the end of each interview, we collected the participants' demographic characteristics. Each interview lasted for between 20 and 90 minutes. The transcripts were manually coded and grouped into categories to explore the initial themes. Data collection and analysis were carried out simultaneously. The analysis of the data was conducted using transcripts in Farsi (the common language of the Iranian people) by the first author. The data were further explored, using content analysis, for the identification of recurring themes. Transcripts were read several times and coded, and emergent themes were identified.

The co-researchers checked the plausibility of the data interpretation and ensured that the qualitative data analysis was systematic and verifiable. To ensure the validity of the analyst interpretations, data source triangulation both across participants and across investigators were employed. More than 25% of the transcripts, codes and categories were rechecked by the study team and a high level of agreements was noted. Disagreements between the researchers were resolved by group discussions.

In addition to using semi-structured interviews, we also employed in-depth interviews to provide further opportunities for the participants to share their untold stories about HIV-related healthcare-seeking experiences. The in-depth and semi-structured interviews used for the collection of data were complementary in method. However, the in-depth interviews shed more light on the consequences of stigma. Some of the participants were more eager to talk about their behavioural and emotional reactions to the HIV-related stigma to the extent that they requested to be scheduled for follow-up interviews.

The Ethics Committee of Tarbiat Modares University approved the study proposal. Participants were informed about the objectives of the research and its confidentiality. Those who agreed to participate in the study were each asked to sign an informed consent document.

## Results

### Socio-demographic characteristics

In all, 69 PLHIV, including 42 (61%) men and 27 (39%) women, took part in this study. Of the total, 49 participants (71%) reported diagnoses by their primary physician as being HIV positive and 20 (29%) reported their health status as full development of AIDS. The median age of patients was 28 and they ranged in age from 21 to 47 years. Most participants were educated at the high school level (from 10 to 12 years of education), while one participant reported no formal education, and four participants had received college educations. Table 1 illustrates the socio-demographic characteristics of participants.

**Table 1:** Socio demographic characteristics of studied patients (n = 69)

Characteristics	Frequency	Percentage
**Gender**		
Male	42	60.9
Female	27	39.1
**Educational level**		
No formal education	1	1.5
Primary	10	14.5
Secondary	22	31.8
Senior high school	28	40.6
College or academic	4	5.8
Unknown	4	5.8
**Marital status**		
Married	28	40.6
Single	26	37.7
Widowed	6	8.7
Divorced	9	13
**Occupation**		
Employed	30	43.5
Unemployed	39	56.5
**Residency**		
Tehran (Iran capital )	59	85.5
Out of Tehran	10	14.5
**Disease phase**		
HIV	49	71
AIDS	20	29

### Themes

While many participants in our study indicated they received quality care and were happy with their healthcare experience, nearly all participants, especially women, reported that they experienced stigma in the healthcare system. Table 2 shows themes that emerged from the in-depth interviews, with participants explaining: (1) causes of stigma; (2) forms of stigma; and (3) consequences of stigma.

**Table 2:** Themes and sub-themes of stigmatization of HIV/AIDS in healthcare system

Themes	Sub-Themes
1. Causes of stigma	a) Healthcare providers fear of being infected
	b) Healthcare providers' views on sexual orientation, drug abuse, prostitution and adultery

2. Forms of stigma in healthcare settings	a) Refusal of care
	b) Sub-optimal care
	c) Excessive precautions and physical distancing
	d) Humiliation or verbal and non-verbal psychological abuse and blaming

3. Consequences of stigma	a) Avoiding or delaying seeking healthcare
	b) Not adhering to HIV treatment
	c) Refusing disclosure of HIV status when seeking care
	d) Feeling undeserving of care and diminished motivation to stay healthy
	e) Using violence and feeling vengeful
	f) Using spiritual healing as a substitute for conventional care
	g) Feeling emotional stress

#### Causes of stigma

Participants perceived that healthcare providers were fearful of becoming infected with HIV and tended to hold negative views and attitudes regarding homosexuality, drug abuse, prostitution and adultery. These views may contribute to HIV-related stigma among healthcare providers.

##### Fear of becoming infected

Participants perceived that healthcare providers' fear of becoming infected was a major cause of stigma against them. For example, a 36-year-old female receptionist spoke of her mistreatment by a provider:

I was hospitalized due to pneumonia. At the time I reported to my doctor that when I menstruated, my bleeding was excessive and did not stop for several days, so my doctor referred me to a gynaecologist for further evaluation. When I arrived at the gynaecology service, the staffs read my medical record and were informed about my history of HIV infection; they hesitated to examine me. I heard my nurse saying to herself that I came here to infect them too.

A 27-year-old unemployed male said:

I have no doubt that one of the nurses in this ward is scared of taking my blood ... One of our main problems among providers, even among physicians, is that they really do not know how HIV is transmitted.

Other participants described their experiences with healthcare providers who would refuse to shake their hands or touch them for fear of becoming infected. A 31-year-old supermarket salesman reported:

When I told my physician about my HIV infection, he quickly sterilized his hands with disinfectant because he had shaken hands with me when I arrived. It immediately affected my spirit. I know my provider has studied this disease and he knows that HIV cannot be transmitted by shaking hands. So why did he act this way?

##### Providers' views on homosexuality, drug abuse, prostitution and adultery

A 32-year-old married housewife said:

Most providers want to know how I was infected. Sometimes they ask this question and even before I answer, they blame me for doing something sinful. I am married, I have never had any relationships out of wedlock. However, a female doctor told me, "Are you sure you have not had a sexual relationship with someone else?" Indeed, she was interrogating me while three other individuals were in the room.

A 25-year-old housewife indicated that "some of the providers think that whoever is infected with the HIV virus is a prostitute or has committed adultery".

In another instance, a 34-year-old restaurant manager remarked:

They (healthcare providers) look at you as if you are a lunatic who deserves to get this horrible disease. I think they assume that you abuse drugs or you are a homosexual.

Similar perceptions were described by other participants who indicated that they felt as though their healthcare providers seemed to believe that whoever was infected with HIV had committed a sin, and as a result, more or less deserved to suffer.

#### Forms of stigma in healthcare settings

Participants in this study indicated at least four major forms of stigma, including: (a) denial of care; (b) sub-optimal care; (c) excessive precautions and physical distancing; and (d) humiliation or psychological abuse and blaming.

##### Denial of care

The majority of participants stated that healthcare providers sometimes refused to provide needed services, and perceived that healthcare providers did so because of their fear of becoming infected with HIV. A 29-year-old female patient, a hairdresser, reported:

Yes, it happened to me when my ovarian cyst ruptured and I needed an immediate operation. The surgeons did not operate on me. They never wanted to. They just came and went. I was examined by several surgeons.

A 25-year-old patient, a housewife, reported that she was denied post-delivery care when the emergency room (ER) doctor became aware of her HIV status. She indicated:

I had excessive bleeding for a few days after I had delivered my baby. I had to go to the ER. I told them that I am HIV positive. The ER doctor told me, "You have excessive bleeding. I cannot examine you. I could be easily infected with the HIV. Look, you have HIV .... Sorry, go somewhere else."

A 27-year-old used car dealer spoke of a similar circumstance in which a nurse had refused to provide services:

I knew a nurse [at the infection ward] who was scared of us. No nurse really wants to take care of PLHIV admitted to this ward. Some of the nurses in particular refused to take blood or give injections to the patients.

With no exception, all patients who needed dental care and had revealed their HIV status to their dentists were directly or indirectly denied dental care. A 31-year-old unemployed male patient reported that on one occasion when he revealed his HIV status to his provider, the dentist immediately asked him to leave the office. A 25-year-old housewife reported visiting a dentist who claimed that his equipment had broken down and he would therefore not be able to provide treatment for her decayed teeth. She perceived his refusal to provide care as a clear demonstration of his unwillingness to serve patients with HIV.

##### Sub-optimal care

A number of patients reported that they had received sub-optimal care because of their HIV status. A 36 year old housewife said:

I was preparing dinner at home when I cut my index finger; the cut was deep and I knew from experience that it would require stitches of some sort, so I went to the local urgent care clinic. I told the doctor that I was HIV positive and then the doctor informed me that I really did not need stitches after all and he wrapped the finger in gauze and tape and sent me home. After a few days the cut became infected and I had to see [the] doctor. This time, [I] went to a different one. This time the doctor had to clean the cut and stitched up my finger."

##### Excessive precautions and physical distancing

A few participants described experiences of healthcare providers taking excessive precautions and keeping a physical distance, including providers who refused to shake their hands or touch them. Participants expressed frustration with their healthcare providers' excessive precautionary measures; many participants remarked that this was confusing to them: they understood that healthcare providers were medical professionals who were supposed to be aware of the HIV disease process, including how the disease was transmitted from one person to another.

Some participants reacted emotionally to the perceived hostility of healthcare providers. A 25-year-old housewife related:

How would you feel about talking about your disease with your doctor when you know that what you have to say will not be considered or even matter to anyone? This hurts your spirit. When the doctor does not care about me, when she/he doesn't communicate with me effectively, what should I expect from other people?

Similarly, a 26-year-old patient, a housewife, said:

Believe [it] or not, there is one nurse in this ward who actually ran away from me. On one occasion, when I approached the nurse's station to ask for something, she told a non-HIV-positive patient, "Cover your mouth, be careful, you do not want to get infected with the HIV." Oh my God, I really do not understand this.

##### Humiliation or verbal and non-verbal psychological abuse and blaming

Many participants from different ages and backgrounds claimed that they had been humiliated, constantly blamed, and verbally abused by their healthcare providers. A 28-year-old seamstress related her experiences in the hospital during the delivery of her child:

I was admitted to a hospital for delivery. When the nurse was informed about my HIV infection, she said, "Why did you become pregnant? Why are you here? There are many children in the orphanage that you could have adopted, instead of becoming pregnant." You see, they give themselves the right to decide for us.

A 31-year-old factory worker reported getting negative non-verbal message from his healthcare provider. He said:

Once I went to the doctor and talked about my CD4 count, he looked at me in a very nasty way. I deeply felt as though he actually blamed me for getting HIV, by the way he looked at me, even though he did not say anything.

In addition, participants reported that they felt as though they were blamed for their infection because of disrespectful behaviours and negative attitudes expressed toward them by their healthcare providers. As mentioned earlier, most participants reported that healthcare providers assumed the right to ask participants how they became infected, even when they were hospitalized for reasons not directly related to their disease. Participants also reported feeling disrespected by healthcare providers who learned of their seropositive status during hospital visits.

#### Consequences of stigma

In-depth interviews with the participants in this study led us to identify the consequences of stigma as: (a) avoiding or delaying seeking healthcare; (b) refusing disclosure of HIV status when seeking care; (c) low self-esteem accompanied by a diminished motivation to stay healthy; (d) using violence and feeling vengeful; (e) using alternative medicine as a substitute for conventional care; and (f) experiencing emotional stress.

##### Avoiding or delaying seeking healthcare and not adhering to HIV treatment

A 25-year-old housewife who needed dental treatment refused to seek care, saying that:

Several of my teeth needed immediate attention. Indeed, occasionally I have pain. However, I am not seeking dental care. I know as soon as I let them know that I am HIV positive, they will tell me, sorry our equipment is broken, come back later ... My husband has stopped taking his medication. His CD4 is less than 800 now. He says, "I cannot handle any more humiliation ... I just want to die."

In a few cases, participants reported that they did not immediately seek treatment upon being diagnosed as seropositive for fear of being mistreated or misjudged by their healthcare providers, especially when their offices were located in the same community. One 28-year-old housewife, who lived in a small town, did not seek care at a local medical centre because she was worried about how health workers would view her and her family:

My husband forced me to go to a medical centre in Tehran, in a big city. My husband did not come with me in the last four or five times that I went to meet with my doctor. I would have liked to have had a consultation in our own town, but my husband and I are not sure how the local doctors will react.

##### Refusing disclosure of HIV status when seeking care (e.g., dental care) and feeling uncomfortable communicating openly with healthcare providers

Perhaps most troublesome to many of the participants was the perception that they could not communicate effectively and honestly with healthcare providers for fear of negative retribution based on previous negative experiences. Participants, as discussed, felt that healthcare providers feared becoming infected from their patients and therefore verbally and emotionally mistreated or denied treatment to patients in their care. In some cases, patients did not disclose their infection at all, while a few patients revealed their HIV status after receiving treatment.

Indeed, a patient indicated that to make sure that his dentist took precautionary measures, he usually told his dentist that he has been diagnosed with hepatitis B or C. A 29-year-old housewife said:

I begged them (dentists) a couple of times to take care of my bad tooth. However, they did not do anything but instead asked me to leave their clinic. After that experience, I did not tell them, dentists or others, any more about my HIV status.

##### Low self-esteem accompanied by a diminished motivation to stay healthy

Several patients claimed that because they had been mistreated by their providers, they had lost their motivation to stay healthy. A 31-year-old male street worker said:

You know, after my doctor mistreated me, I left his office angry. I told myself that my life is over; I really do not have any reason to take care of myself. This is the last page of my life; my life no longer has any meaning.

A 22-year-old unemployed male indicated:

My own doctor has no respect for me. This makes me very upset. I felt as though I do not deserve any care. I am worthless. I do not know ... maybe I should go somewhere else, another country.

##### Using violence and feeling vengeful

Several patients, particularly female patients who claimed that they had been inadvertently infected through their husbands, expressed strong vengeful feelings toward healthcare providers who openly blamed or mistreated them. A 25-year-old married patient, a housewife, said:

Oh my God, their (providers') behaviours and attitudes toward me made me so upset ... I really hate them. I do not understand. They are educated ... they should know better. Sometimes I feel that I should take revenge.

A 35-year-old male factory worker said:

I really wanted to take revenge by using the same syringe that they had used to take blood from me.

##### Using alternative medicine as a substitute for conventional care

Even though none of our participants reported explicitly using alternative medicine as a substitute for conventional care, a few participants admitted that they had been using spiritual healing by travelling to Islamic holy land and to the tombs of Islamic leaders to get cured, partly because of the stigma they had experienced in the healthcare system.

##### Feeling emotional stress

Participants in our study indicated that they had had numerous interactions with their healthcare providers during the course of their disease, and that these interactions caused them tremendous emotional stress. Participants reported avoiding the use of healthcare in general, including non-HIV-related care (e.g., dental care) within their communities, and especially in their neighbourhoods. Also, participants reported that they did not expect to receive support if they chose to disclose their HIV status; this ultimately led to emotional stress among our participants, who reported experiencing HIV-related stigma.

Several patients in our study indicated that they had felt deeply depressed when they were denied care or received sub-optimal care by their health care providers. Indeed, one of the patients indicated that her doctors' and other healthcare providers' attitudes and behaviours toward her made her feel "paranoid"; she claimed that she had every right to be paranoid.

#### Supportive care and non-stigma quality care

It is important to note that a vast majority of participants in this study who reported being stigmatized clearly indicated that discrimination by some healthcare providers should not be generalized to all of them. In addition to receiving sub-optimal care or being denied care, as well as being blamed for their HIV status, many participants believed that they had received strong supportive care from many other healthcare providers. A 39-year-old female airport custodian said:

I was so scared when I learned that I was infected with the HIV, I had lost all hope. I was devastated. However, my doctor changed my life. He helped me to think differently. I really felt calm after talking to him.

A 22-year-old housewife talked about the supportive role of physicians during her pregnancy:

I was a pregnant woman when I was diagnosed as HIV positive, so I became disappointed. I thought both my baby and I would die at any moment. When I came to [my] doctor [at the] prenatal clinic, they encouraged me and said that I would not die and said I could live well. I said again, I am going to die. In response, different health workers encouraged me and said if I took precautionary measures, I would not die. Now I have become somewhat hopeful about living.

Finally, a few participants in this study mentioned that when they were referred by the HIV clinic for a certain type of care, they felt less discrimination and experienced more quality in their care. For example, a 32-year-old housewife described her experience visiting a dentist to whom she had been referred:

I had a toothache two years ago. I should have gotten my tooth treated, but [whenever] I called a dentist office, they did not admit me. But after a while, the same dentist whom I visited a long time ago accepted to treat my tooth. This was a change in their attitudes, because the HIV clinic had introduced me to the dentist.

## Discussion

Prior to elaborating on our findings, it is important to note that participants for this study were selected from two HIV clinics in central Tehran, and therefore the results of this study could not be generalized to all Iranian PLHIV. Patients who had not visited these treatment sites were not included in the present research.

Our data shows that nearly all participants in this study reported experiencing stigma and discrimination by their healthcare providers in a variety of contexts. While previous studies demonstrate that most Iranian healthcare providers report fairly positive attitudes towards PLHIV [35], our participants' experiences and perceptions of the attitudes and behaviours of healthcare providers tell a different story. Therefore, it is imperative to engage PLHIV in designing effective interventions targeting healthcare providers.

### Causes of stigma

Participants in this study perceived that healthcare providers' fear of becoming infected with HIV, coupled with religion- and value-based assumptions about PLHIV, led to high levels of stigma. The perceived negative attitudes of healthcare providers demonstrated in this study are supported by previous studies in different countries [39-43].

PLHIV who participated in this study often pointed to the concept of religious sins (*gonah*) as they relate to HIV transmission and stigma. Participants felt that providers linked HIV-positive status to drug addiction, prostitution, adultery, alcoholism and homosexuality, all of which are seen as religious sins. Participants clearly perceived that religious-based values led to negative attitudes toward PLHIV. However, it is important to note that none of the participants indicated that healthcare providers who seem more religious had exercised more discrimination against them. Further studies are needed to investigate the impact of religiosity on attitudes toward providing healthcare to PLHIV in Muslim communities, or what it means to be an HIV-positive Muslim, and to live in an Islamic country where practically all healthcare providers are Muslim.

The finding that social stigma is related to the suspicion that HIV has been transmitted via drug addiction, prostitution, adultery and/or homosexuality is ubiquitous in academic literature. Islamic law strongly condemns all of these behaviours and/or lifestyles with harsh punishment, and likely exasperates the stigma against PLHIV in healthcare settings. For example, Islamic law deems prostitution and adultery to be great sins punishable by stoning.

It is important to consider what should be done in future research, programming and/or policy to change this situation, and to examine how efforts made in western countries to reduce stigma may be applied in Iran. Extensive qualitative research is needed to answer these questions. Some solutions may be suggested within Islamic law itself, which equally condemns discrimination against patients. Investigating Islamic literature and instruction, as well as requesting official public statements from spiritual leaders (*ayatollahs*) regarding stigma and discrimination in healthcare settings, should be used in educational intervention programmes targeting healthcare providers.

Participants in this study perceived that healthcare providers feared that providing treatment to serpositive patients would make them susceptible to HIV infection, which led to irrational and discriminatory treatment of PLHIV. This practice persists among healthcare providers in Iran despite strong evidence that little or no risk of HIV transmission for healthcare providers exists with basic precautionary practices in place [44]. Consistent with these results, other studies report misconceptions about modes of HIV transmission that lead to refusing care to HIV patients [45-48].

However, two interesting findings of this study warrant further discussion: (1) participants experienced higher quality care when visiting healthcare providers to whom they were referred directly by an HIV clinic; and (2) participants consistently experienced poor quality care from dentists. Participants may have more positive experiences with providers to whom they are referred by HIV clinics because these healthcare providers presumably receive a large number of HIV patients through the referral process, and therefore may be more likely to be informed, educated and prepared to provide care to PLHIV.

Additionally, official referrals by HIV clinics to healthcare providers may carry more formal accountability than patient-initiated requests for care. Regardless of the reasons, we suggest that HIV clinics develop an official referral office for PLHIV, and further, that PLHIV should be encouraged to request all types of referrals from HIV clinics, particularly for non-HIV-related care.

The second finding, that dentists have greater fear of occupation-related HIV risk than physicians and other healthcare providers, has been supported by Jovic-Vranes *et al *(2006) [49]. Another study revealed that Iranian physicians, when compared with nurses and lab technicians, were less afraid of becoming infected and more interested in caring for PLHIV [35]. One plausible explanation is that physicians might be more knowledgeable regarding modes of HIV transmission. However, more research is needed in order to understand the impact of HIV and AIDS knowledge on the attitudes and behaviours of healthcare providers toward patient care [35].

Previous studies have emphasized education of clinical staff through: role modelling, discussions and counselling about modes of HIV transmission; universal precautions; decreasing discrimination; and consideration of human rights [50]. Educational programmes need to provide healthcare workers with complete information about how HIV is and is not transmitted, and how practicing universal precautions can allay their fears [23]. Researchers recommend well-coordinated, continuing education about HIV and AIDS for all categories of healthcare providers [51].

Strategies to address healthcare providers' concerns, such as the development of interventions to promote occupational safety, are likely to ameliorate the discrimination experienced by PLHIV when accessing healthcare services [52]. In particular, we suggest that a specific educational programme be designed for dentists, and that this programme be included as part of license renewal training and education.

### Forms and consequences of stigma

PLHIV reported being refused care or receiving sub-optimal care; this is consistent with previous study findings [43,53,54]. Participants feeling blamed for their infection while seeking care is also echoed in reports from previous studies of PLHIV [44,55]. PLHIV must negotiate feeling blamed for their infection in the context of receiving treatment by the medical community, as well as in other areas of their life [44].

However, it is important to examine this rejection and blaming within the context of the patient-provider relationship in Iranian culture. This relationship is traditionally based on compassion, friendliness, altruism and mutual trust. In Iranian Shi'a-Islamic culture, the physician is *mahram*. In this context, *mahram *means that one is allowed to expose his or her body to someone who is not his or her spouse, which is otherwise condemned by Islamic law. Iranian Islamic culture traditionally considers the most prestigious professions to be the study of religion and/or the study of the body. Indeed, clergy and physicians provide care for an individual's mind (spiritual) and body, respectively.

Given this context, when a healthcare provider, who is culturally recognized as a patients' confidante and trustee under *mahram*, rejects or mistreats his or her patient, the patient faces an extremely humiliating situation that is far more degrading than being rejected by any other service. This rejection likely causes psychological distress with negative health consequences. In our study, such consequences included participants avoiding or delaying seeking healthcare, refusing disclosure of HIV status when seeking healthcare, and using spiritual healing as a substitute for conventional care.

Avoiding and delaying seeking healthcare in local settings by participants who had commuted from smaller cities to the two HIV clinics in Tehran was particularly alarming. Several participants clearly reported that they had not sought healthcare in their own towns because they did not want to be mistreated or misjudged by local healthcare providers. This suggests heavier stigma and discrimination in small and local healthcare settings. Therefore, it is imperative that the Iranian healthcare system combat stigma by focusing on educational training and policy at the national level that particularly targets both HIV clinics and regular healthcare facilities.

In addition to behavioural reactions to perceived acts of stigma, emotional reactions included developing low self-esteem, accompanied by a diminished motivation to stay healthy, feeling angry and vengeful, and being emotional stressed. Other researchers have reported similar results. A recent study examined the experiences of individuals living with HIV who resided in an area with low HIV prevalence, and reported that the reactions to perceived acts of stigma and discrimination by PLHIV from remote areas included anger, shame, social isolation and self-advocacy [42].

In a recent investigation of Iranian healthcare performance on HIV and AIDS, NBC: Around the World reported, "In a region where other Muslim governments ignore the AIDS epidemic, quarantine HIV-infected people or preach abstinence as the only solution, Iran's approach is fairly progressive. Iran's AIDS programme melds up-to-date programmes and research with deep-rooted religious values" [56]. However, HIV-positive Iranian persons, to a large extent, face shame and isolation, and often do not seek medical care in a timely manner.

## Conclusions

Even though the Islamic Republic of Iran has been a leading country in HIV prevention and treatment in the Middle East [30,56], the Iranian healthcare system has yet to combat stigma in both HIV and other health facilities. Doing so will truly make Iran the leading Muslim country in the region in its effort to stop the spread of HIV. As long as those who are infected remain hidden and do not seek care at medical facilities in a timely manner, efforts to stop the spread of HIV are inadequate.

Iranian healthcare authorities must show support and designate resources to stigma-reduction activities nationally [23]. An integrated approach to healthcare based on a human rights framework, grounded in community realities and delivered in partnership with PLHIV offers the most viable approach to overcoming the deficiencies of HIV and AIDS care in the healthcare system [57].

## Competing interests

The authors declare that they have no competing interests.

## Authors' contributions

FR conceived the study and participated in the design, data collection and analysis. SN conceived the study, took part in coordination and helped to draft the manuscript. MB participated in the design of the study and preparation of the manuscript. FAm participated in the design of the study and interpretation of data. FAa participated in the design of the study and interpretation of data. EH participated in the interpretation of data. SST participated in the design of the study and interpretation of data. All authors read and approved the final manuscript.
